# Early intrahepatic duct defects in a cystic fibrosis porcine model

**DOI:** 10.14814/phy2.14978

**Published:** 2021-07-20

**Authors:** Keyan Zarei, David K. Meyerholz, David A. Stoltz

**Affiliations:** ^1^ Department of Internal Medicine Roy J. and Lucille A. Carver College of Medicine University of Iowa Iowa City IA USA; ^2^ Department of Biomedical Engineering Roy J. and Lucille A. Carver College of Medicine University of Iowa Iowa City IA USA; ^3^ Department of Pathology Roy J. and Lucille A. Carver College of Medicine University of Iowa Iowa City IA USA; ^4^ Department of Molecular Physiology and Biophysics Roy J. and Lucille A. Carver College of Medicine University of Iowa Iowa City IA USA; ^5^ Pappajohn Biomedical Institute University of Iowa Iowa City IA USA

**Keywords:** CFTR, cholangiocytes, cystic fibrosis, intrahepatic bile ducts, organoids

## Abstract

Hepatobiliary disease causes significant morbidity and mortality in people with cystic fibrosis (CF), yet this problem remains understudied. Previous studies in the newborn CF pig demonstrated decreased bile flow into the small intestine and a microgallbladder with increased luminal mucus and fluid secretion defects. In this study, we examined the intrahepatic bile ducts of the newborn CF pig. We assessed whether our findings from the gallbladder are present elsewhere in the porcine biliary tract and if CF pig cholangiocytes have fluid secretion defects. Immunohistochemistry demonstrated apical CFTR expression in non‐CF pig intrahepatic bile ducts of a variety of sizes; CF pig intrahepatic bile ducts lacked CFTR expression. Assessment of serum markers did not reveal significant signs of hepatobiliary disease except for an elevation in direct bilirubin. Quantitative histology demonstrated that CF pigs had smaller bile ducts that more frequently contained luminal mucus. CF intrahepatic cholangiocyte organoids were smaller and lacked cAMP‐mediated fluid secretion. Together these data suggest that cholangiocyte fluid secretion is decreased in the CF pig, contributing to structural changes in bile ducts and decreased biliary flow.

## Graphical abstract

Using a porcine CF model, we discovered that intrahepatic ducts in newborn CF pigs have structural changes and are commonly plugged with mucus. CF intrahepatic cholangiocyte organoids had impaired cAMP‐mediated fluid secretion. Together these data suggest that impaired cholangiocyte fluid secretion contributes to structural changes in bile ducts, bile duct plugging, and decreased biliary flow.

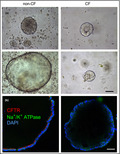

## 1 INTRODUCTION

CF liver disease (CFLD) is the third‐leading cause of mortality in people with CF (Registry, 2018). Up to a third of people with CF develop CF‐related hepatobiliary disease (Colombo et al., 2002; Lamireau et al., 2004). Recent studies suggest that the cumulative incidence of CFLD increases as people with CF get older (Boelle et al., 2019; Toledano et al., 2019). CF therapies have increased the median age of survival to 47 years of age (Foundation CF, 2019). As the CF population gets older, the prevalence of CFLD may increase. Thus, there is a need to better understand the pathogenesis of CFLD.

Within the liver, CFTR is only expressed in the bile ducts (Cohn et al., 1993). In humans, there are a variety of different manifestations of CF hepatobiliary disease; most commonly CFTR dysfunction associates with focal biliary cirrhosis (Blanc & Di Sant'Agnese, 1956; Colombo, 2007; Colombo et al., 2002; Leung & Narkewicz, 2017; Oppenheimer & Esterly, 1975a, 1975b; Vawter & Shwachman, 1979). In severe CFLD cases, multilobular fibrosis or portal hypertension develops requiring liver transplantation (Colombo et al., 2002; Lindblad et al., 1999). It is unclear why only some people with CF develop hepatobiliary disease or why there is such a broad spectrum of CFLD; furthermore, the pathogenesis of CFLD remains unclear (Debray et al., 2011).

Mouse models have contributed to our understanding of CFLD. Alterations in bile acid composition have been identified in CF mouse models (Bodewes et al., 2015b; Debray et al., 2012). Work by Strazzabosco et al. identified CFTR as a regulator of innate immunity and CFTR loss led to an exaggerated TLR4 response to endotoxin (Fiorotto et al., 2011). This abnormal innate immune response caused cytokine production and immune cell recruitment, contributing to the development of CFLD in mice (Fiorotto et al., 2016). These mouse studies suggest that CFLD requires a “second‐hit.” While this is interesting, there are some limitations with CF mouse models. For instance, without aging or induced colitis, *CFTR* ^−/−^ mice develop mild to no hepatobiliary disease compared to humans (Fiorotto et al., 2019; Olivier et al., 2015). Thus, translating results from mouse studies to human CFLD may be challenging.

The CF pig has signs of hepatobiliary disease at birth and recapitulates disease seen in humans (Meyerholz et al., 2010; Rogers et al., 2008; Uc et al., 2012). For example, some newborn CF pig livers have biliary proliferation, fibrosis, and inflammation; and multilobular cirrhosis can be observed in older CF pigs (Meyerholz et al., 2010; Rogers et al., 2008; Stoltz et al., 2013). The exact pathogenesis contributing to these changes is not clear. In newborn CF pigs, we previously found reduced bile flow into the small intestine and that the CF pig gallbladder was obstructed by mucus without signs of infection, inflammation, or changes in mucin expression (Uc et al., 2012; Zarei et al., 2020). In the extrahepatic tract, we also showed that CF pig gallbladder organoids lacked cAMP‐mediated fluid secretion (Zarei et al., 2020). Our rationale for this study was to further examine the newborn CF pig liver and assess if fluid secretion defects and mucus changes contribute to early CF disease in the intrahepatic tract. We hypothesized that like the newborn CF pig gallbladder, the intrahepatic bile ducts would demonstrate mucus changes and fluid secretion defects without other signs of hepatobiliary disease.

## 2 MATERIALS AND METHODS

### 2.1 CF pig model

The development of the CF pig model has been previously reported (Rogers et al., 2008). Animals were purchased from Exemplar Genetics (Sioux City, IA). All protocols were approved by the University of Iowa Institutional Animal Care and Use Committee (IACUC). Newborn (within the first 24 h of life) *CFTR* ^+/+^ and *CFTR* ^−/−^ pigs (henceforth referred to as non‐CF and CF pigs, respectively) were sedated with ketamine/xylazine (Akorn) and euthanized with phenobarbital sodium/phenytoin sodium (Euthasol; Virbac).

### 2.2 Immunohistochemistry

Immunohistochemistry studies were performed by the University of Iowa Comparative Pathology Laboratory (Meyerholz et al., 2016). Briefly, the liver and gallbladder from newborn piglets were excised and fixed in 10% normal‐buffered formalin (room temperature, 1 h). Tissues were embedded in paraffin, serially sectioned using a microtome (~4 µm), and dehydrated through a sequence of xylene/alcohol baths. Subsequently, antigen retrieval was performed using the NxGen Decloaking Chamber^TM^ (Biocare Medical) with a citrate buffer (pH 6.0, 110°C, 15 min). Endogenous peroxidase activity was quenched (hydrogen peroxide 3%, 8 min), endogenous avidin/biotin was blocked (Avidin/Biotin Blocking Kit, Vector Laboratories, Inc.), and nonspecific background was blocked (equine serum, 5% in 1x Dako Buffer). CFTR immunohistochemistry was performed with mouse anti‐CFTR 769 (1:1200, 60 min; CFF/UNC), generously supplied by Dr. John Riordan, University of North Carolina—Chapel Hill and the Cystic Fibrosis Foundation Therapeutics. The anti‐CFTR antibody was validated based on cellular localization, absent signal in CFTR‐deficient tissue, and previous studies (Zarei et al., 2020). Secondary Ab (1:200, 30 min; Vector Biotinylated Anti‐Mouse IgG) was then applied followed by Vector ABC Reagent (30 min, Standard VECTASTAIN^®^ Elite^®^ ABC Kit, Vector Laboratories, Inc.) and chromogen (room temperature, DAB plus for 5 min followed by DAB Enhancer for 3 min). Positive and negative controls (pancreatic duct cells and hepatocytes, respectively) were used during staining to validate staining specificity. Tissues were counterstained with Harris hematoxylin (1 min, Surgipath, Leica Microsystems, Inc.). Slides were blued in Scott's Tap water, dehydrated through a sequence of alcohol/xylene baths, and subsequently coverslipped. High‐resolution images of the slides were acquired with the 20× objective at 0.24 μm/pixel resolution by the Panoramic 1000 slide scanner (3DHISTECH).

### 2.3 Histopathology and quantitative histology

Tissues were fixed, embedded, and processed as described for immunohistochemistry. The Comparative Pathology Laboratory (University of Iowa) completed histochemical staining with amylase‐pretreated Periodic acid‐Schiff (dPAS). High‐resolution images of the slides were acquired with the 20× objective at 0.24 μm/pixel resolution by the Panoramic 1000 slide scanner (3DHISTECH). Tissue sections were visualized using the 3DHISTECH CaseViewer software (3DHISTECH) to quantify histological features of all tissue specimens. To provide a standardized sampling for measurements, the right medial liver lobe was sectioned containing the gallbladder. A liver parenchyma area of at least 30 mm^2^ was evaluated and all ducts within the liver section that had a discernable lumen were quantified. Quantitative (duct diameter and lumen diameter) and semi‐quantitative (mucus scoring) measures were performed in a manner blinded to the group to avoid bias (Meyerholz & Beck, 2018). To account for different sectioning planes, the minor diameter was measured for the duct and lumen diameters. We studied mucus using the dPAS stain (Meyerholz et al., 2018a). Ordinal scoring of mucus had 4 levels: 0–no dPAS staining present in duct, 1–dPAS staining present intracellularly or on the cell surface, 2–dPAS staining present in lumen, 3 – lumen obstructed with dPAS material, bile, or cellular material.

### 2.4 Newborn non‐CF and CF serum analysis

Blood was collected from newborn pigs within the first 24 h of life. Subsequently, the serum was transferred into specialized serum‐separation collection tubes. The University of Iowa Hospitals and Clinics Diagnostic Laboratories performed serum‐based tests assaying liver transaminases (alanine transaminase, aspartate transaminase), bilirubin (total and direct), gamma glutamyl‐transferase, and alkaline phosphatase levels.

### 2.5 Generation of intrahepatic pig biliary organoids

Methodology for the organoid model was adapted from a published protocol for biliary organoids (Sampaziotis et al., 2017). Liver samples were placed in cold William's E media supplemented with nicotinamide (10 mM, Sigma), sodium bicarbonate (17 mM), 2‐phospho‐L‐ascorbic acid trisodium (0.2 mM, Sigma), sodium pyruvate (6.3 mM, Sigma), glucose (14 mM), HEPES (20 mM), dexamethasone (100 nM), insulin–transferrin–selenous acid (ITS) premix (1:100, Corning), and penicillin–streptomycin (100 U/ml; 100 µg/ml, Gibco). This media will henceforth be referred to as supplemented William's E media. Tissues were kept at 4°C for less than 24 h until the sample could be processed.

Approximately a 2 × 2 × 2 mm section of liver was removed, minced using sterile scalpels in William's E media containing collagenase (1 mg/ml, Sigma Aldrich), and incubated for 1 h at 37°C. The digestion solution was then mechanically disrupted and strained over a 70 µm cell strainer. Collagenase was neutralized by adding William's E media with fetal bovine serum for a final concentration of 2% fetal bovine serum by volume. Cells were centrifuged down at 440 *g* for 5 min. The cell pellet was washed with supplemented William's E media and centrifuged down again at 440*g* for 5 min. The resulting cell pellet was resuspended in supplemented William's E media containing the following growth factors: 500 ng/ml of human recombinant R‐spondin 1 (R&D) and 40 ng/ml of human epidermal growth factor (R&D). This growth factor‐containing media is referred to as biliary organoid media. For the initial plating of the cells, 10 μM Y‐27632 dihydrochloride (Tocris) was also added to the media but was not included for subsequent media changes. The cell pellet was mechanically dissociated so that there were cell clusters of approximately 5–20 cells and resuspended in biliary organoid media. Matrigel (Corning) was added to the cell suspension (Matrigel‐cell suspension ratio by volume = 2:1) and the final mixture was plated in prewarmed 6‐well or 24‐well culture plates in small, 10–20 µl drops. The plates were inverted and incubated for 10–20 min at room temperature and then for another 30–40 min at 37°C. Four hundred microliters (24‐well) or 1 ml (6‐well) of prewarmed biliary organoid media was then added to each well. Media was changed every 3–4 days. Organoids were passaged approximately once every 2–3 weeks: organoids were mechanically dissociated from Matrigel using cold William's E media, centrifuged at 440*g* for 5 min, and re‐plated at a dilution of 1:5.

### 2.6 Organoid swelling assay

Methodology for organoid swelling was adapted from an existing protocol (Boj et al., 2017). All experiments were performed on the Zeiss LSM‐880 multiphoton confocal microscope with temperature control (37°C) and humidity chamber. Intrahepatic organoids were plated in 96‐well plates in 3 µl drops (1:2 biliary organoid media to Matrigel) 1–2 days prior to imaging and supplemented with 100 µl of biliary organoid media with 10 μM Y‐27632 dihydrochloride. On the day of the experiment, one vial of calcein green (50 μg; Invitrogen, Thermo Fischer) was thawed and dissolved in 5.1 μl of DMSO. The resuspended calcein green (2.5 μl) was added to 580 μL of organoid media. 10 μL of this final solution was then added to each well for imaging and allowed to incubate for 30 min. Approximately 30 min prior to the experiment, media was aspirated and replaced with Krebs–Ringer solution [118.9 mM NaCl, 25 mM NaHCO_3_, 1.2 mM CaCl_2_, 1.2 mM MgCl_2_, 2.4 mM K_2_HPO_4_, 0.6 mM KH_2_PO_4_, and 5 mM dextrose in 5% CO_2_ (vol/vol), pH = 7.4]. Baseline measurements were obtained. Forskolin (final concentration =10 μM) or DMSO (final dilution = 1:1000) was added to the wells and organoids were monitored for 1 h with an image acquisition interval of 5 min. Segmentation and area measurement for each well were performed through the Zen software (Zeiss) and reported as a total area per well.

### 2.7 Organoid immunocytochemistry

Organoid immunocytochemistry was based on (Dekkers et al., 2019). Biliary organoids were mechanically dissociated from the Matrigel, washed, and fixed in 4% PFA for 15 min at 4ºC. Organoids were kept in suspension at 4ºC while they were permeabilized in 0.3% Triton for 1 h and blocked in Superblock (Thermo Fisher) with 4% normal goat serum for 48 h. Organoids were then incubated with primary antibodies overnight: mouse anti‐CFTR (clone 769) (1:100 dilution, University of North Carolina—Chapel Hill and the Cystic Fibrosis Foundation Therapeutics) and rabbit anti‐Na^+^/K^+^‐ATPase (clone EP1845Y) (1:100 dilution, Abcam). The anti‐CFTR antibody was validated based on cellular localization, absent signal in CFTR‐deficient tissue, and previous studies (Zarei et al., 2020). The anti‐Na^+^/K^+^‐ATPase was validated based on cellular localization and previous studies (Nieto‐Torres et al., 2014). Organoids were then washed with PBS and incubated overnight with goat anti‐mouse and goat anti‐rabbit secondary antibodies conjugated to Alexa‐Fluor 488 and Alexa‐Fluor 568 (Molecular Probes/Invitrogen), respectively. Secondary‐only controls were used during staining to identify any non‐specific staining. Organoids were then washed in PBS, mounted with VECTASHIELD plus DAPI (Vector Labs), plated on chambered coverglass (Lab‐Tek), and visualized with a Zeiss LSM‐880 multiphoton confocal microscope 40x water lens.

### 2.8 Statistics

All statistics were conducted using GraphPad Prism software unless stated otherwise. Non‐parametric *t*‐tests (Mann–Whitney) were conducted when appropriate. *p* < 0.05 was considered statistically significant.

## 3 RESULTS

### 3.1 CF pig intrahepatic bile ducts lack CFTR

**FIGURE 1:**
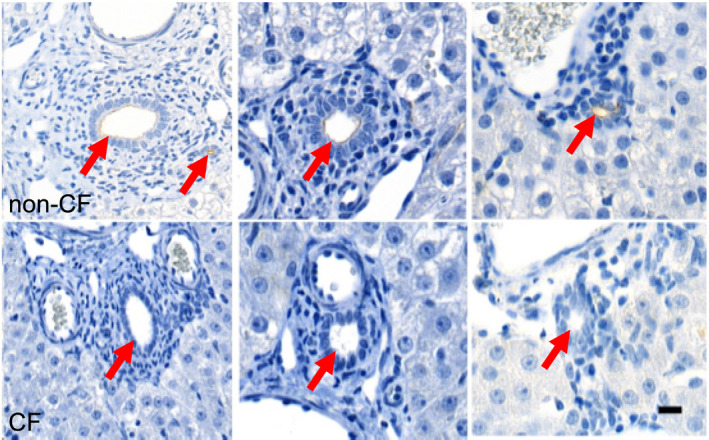
Newborn pig intrahepatic bile ducts express apical CFTR. Examples of apical CFTR in the newborn pig intrahepatic biliary tract detected by immunohistochemistry (brown) for non‐CF and CF, top and bottom, respectively (scale bar = 20 μm). Red arrows indicate bile ducts

We previously showed that CFTR is expressed in the pig gallbladder (Zarei et al., 2020). Studies with human tissue have demonstrated CFTR expression in the intrahepatic bile ducts (Cohn et al., 1993). Immunohistochemistry studies of non‐CF pig livers revealed apical CFTR staining in different sized intrahepatic bile ducts; this staining was present in 100% of non‐CF intrahepatic bile ducts and absent in CF pig samples (Figure 1). No other cell type within the non‐CF pig liver demonstrated positive CFTR staining. Thus, in newborn pigs, CFTR expression within the liver localizes to the apical membrane of the biliary tract.

### 3.2 CF pigs do not demonstrate significant alterations in serum markers of liver disease

**FIGURE 2:**
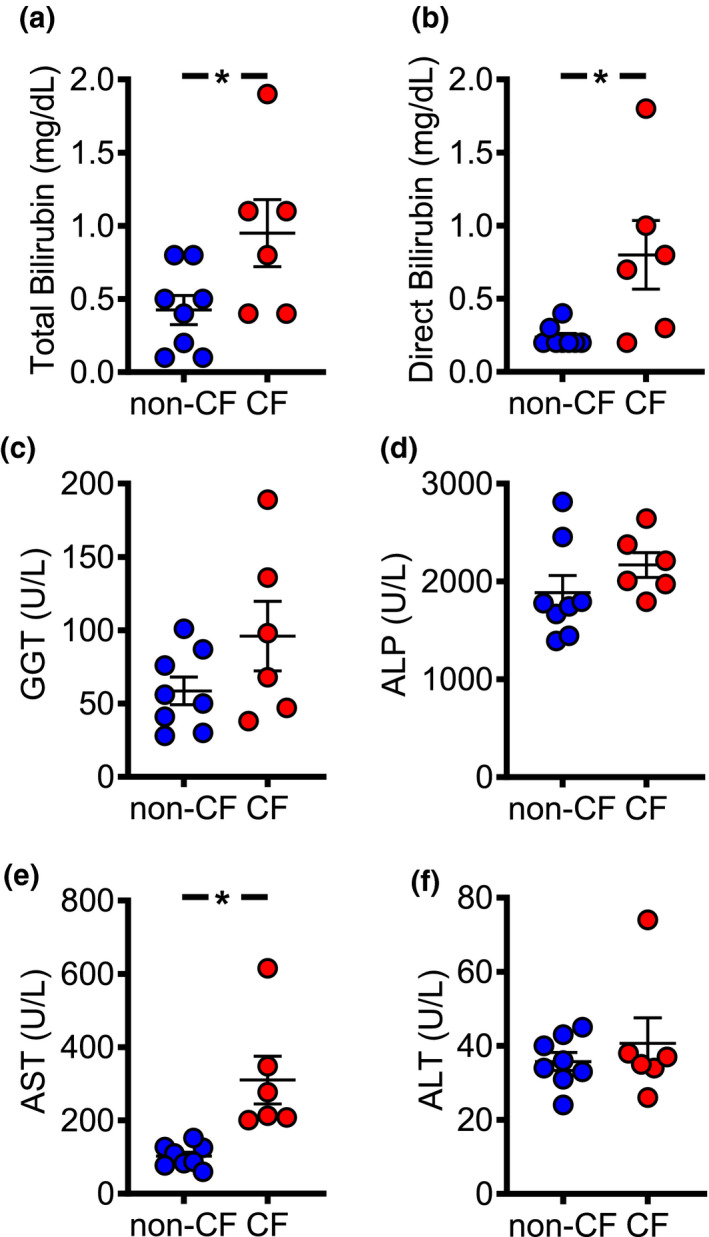
Serum markers of hepatobiliary dysfunction in newborn CF pigs. Serum biomarkers associated with biliary dysfunction include: (a) total bilirubin, (b) direct bilirubin, (c) gamma‐glutamyl transferase, (d) alkaline phosphatase, (e) AST, and (f) ALT. Each symbol represents a serum sample from one animal [non‐CF *n* = 8 (4 male, 4 female), CF *n* = 6 (3 male, 3 female)]. Bars represent mean ± SEM. *p < 0.05 using Mann–Whitney test

We asked whether CFTR loss caused hepatobiliary dysfunction that could be detected in the blood. Newborn CF pigs had elevated total and direct bilirubin serum levels compared to non‐CF (Figure 2a,b), but similar indirect bilirubin levels between genotypes (data not shown). Other serum markers associated with biliary disease, alkaline phosphatase, and gamma‐glutamyl transferase tended to be higher in CF relative to non‐CF pigs but did not reach statistical significance (Figure 2c,d). Aspartate aminotransferase (AST), but not alanine aminotransferase (ALT), levels were elevated in the CF pig (Figure 2e,f). These findings suggest that newborn CF pigs do not have significant changes in serum‐based hepatobiliary markers.

### 3.3 **The newborn CF pig intraheptic biliary** **tree has smaller duct lumens**

**FIGURE 3:**
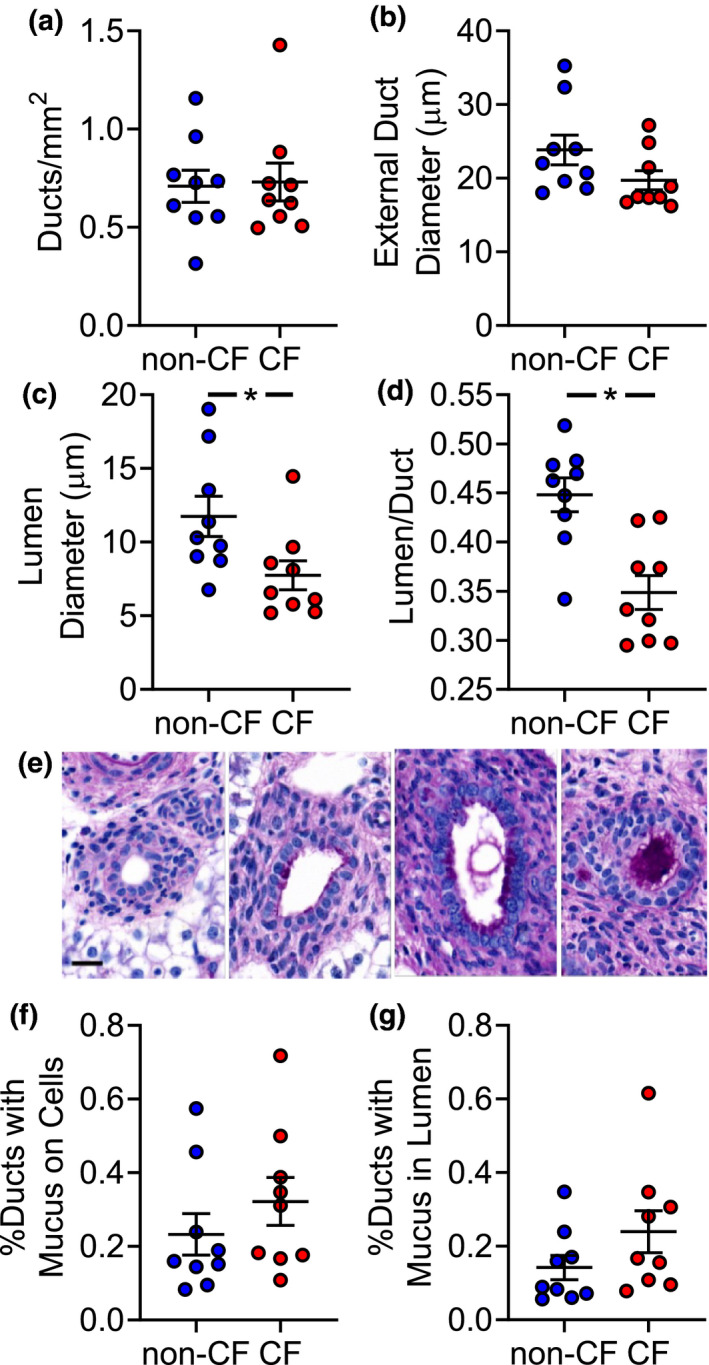
Changes in the porcine intraheptic biliary tract with CFTR loss. Quantitative histological measures of bile ducts include: (a) ducts per area of liver, (b) average duct diameter, (c) average duct lumen diameter, and (d) lumen‐to‐duct diameter ratio. (e) dPAS representative images of mucus scoring in intrahepatic bile ducts (from left to right): no mucus in lumen, epithelial‐associated mucus, luminal mucus without obstructed lumen, and luminal mucus with obstructed lumen (scale bar = 20 μm). (f) Total (epithelial and luminal) mucus scoring and (g) luminal mucus scoring. Each symbol represents measurements from one animal [non‐CF *n* = 9 (6 male, 3 female); CF *n* = 9 (3 male, 6 female)]. Bars represent mean ± SEM. *p < 0.05 using Mann–Whitney test

At birth, all CF pigs have microgallbladders that are occluded with mucus (Meyerholz et al., 2010). To determine if similar changes are present in intrahepatic bile ducts with CFTR loss, we conducted a quantitative histological study of newborn pig intrahepatic bile ducts. CF pig livers contained similar numbers of bile ducts per area and these bile ducts trended toward smaller external duct diameters compared to non‐CF (*p* = 0.07) (Figure 3a,b). However, CF intrahepatic bile duct lumen diameters were smaller than non‐CF (Figure 3c,d). We also developed a quantitative scoring system to assess mucus changes within the CF pig intrahepatic biliary tract (Figure 3e). Compared to non‐CF, a greater portion of CF intrahepatic bile ducts tended to contain mucus, either associated with the epithelium or in the duct lumen; however, this did not reach statistical significance (Figure 3f,g). Thus, CF pig intrahepatic bile ducts are smaller and tend to contain greater mucus than non‐CF.

### 3.4 CF intrahepatic biliary organoids demonstrate morphological defects and lack forskolin‐induced swelling

**FIGURE 4:**
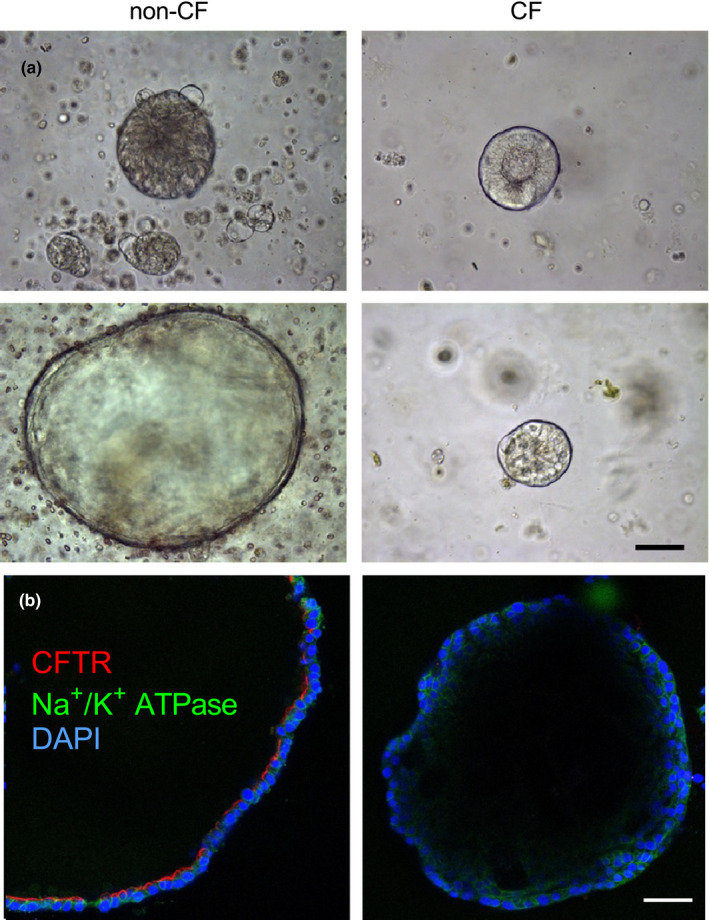
CF porcine biliary organoids demonstrate structural defects. (a) Brightfield images of non‐CF (left) and CF (right) organoids after 1 day (top) and 4 days (bottom) in culture (scale bar = 50 μm). (b) Immunofluorescence showing apical CFTR (red) in non‐CF and CF porcine biliary organoids. Green = Na^+^/K^+^ ATPase. Blue = DAPI (scale bar = 50 μm)

**FIGURE 5:**
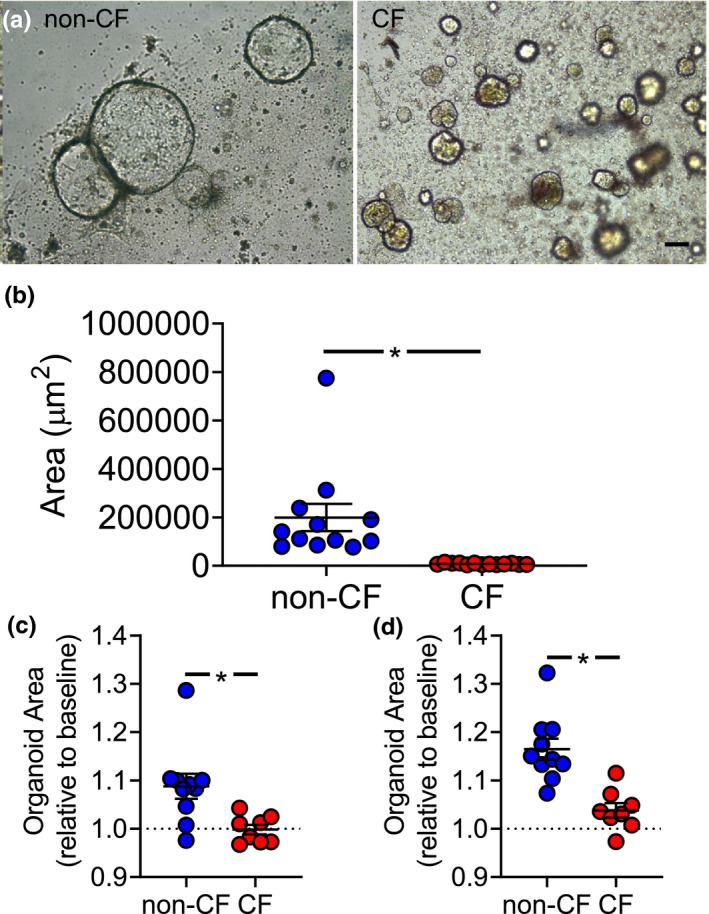
CF porcine biliary organoids are smaller at baseline. (a) Brightfield images of non‐CF (left) and CF (right) organoids after 9 days in culture (scale bar = 100 μm). (b) Organoid area for non‐CF and CF porcine biliary organoids on day 9 of culture [non‐CF *n* = 12 (8 male, 4 female), CF *n* = 13 (5 male, 8 female)]. (c) Fold change of whole organoid area post‐incubation in 10 µM forskolin for 30 min. Values are normalized to organoid response to vehicle (DMSO). (d) Maximal swelling achieved, on average, by non‐CF and CF organoids prior to swelling‐induced rupture. Each symbol represents average organoid area from one animal [non‐CF *n* = 10 (5 male, 5 female), CF *n* = 8 (5 male, 3 female)]. Organoid swelling assays were performed after 1–2 days of culture. Bars represent mean ± SEM. *p < 0.05 using Mann–Whitney test

We previously demonstrated reduced secretin‐stimulated bile flow into the intestine of newborn CF pigs (Uc et al., 2012). We hypothesized that this defect was due, in part, to reduced cholangiocyte fluid secretion. To test this hypothesis, we created organoids from pig intrahepatic cholangiocytes (Tysoe et al., 2019). Within 4 days, organoids formed from liver tissue (Figure 4a). Non‐CF organoids demonstrated a large central lumen surrounded by a layer of cells, while CF organoids did not form a substantial lumen (Figure 4a). Immunofluorescence studies in non‐CF organoids showed CFTR staining along the epithelial surface facing the lumen. This staining pattern confirmed apical‐in organoid polarity but also that these organoids contain cholangiocytes (Figure 4b). CF organoids did not demonstrate CFTR staining (Figure 4b). After 9 days in culture, non‐CF organoids were larger than CF under unstimulated conditions (Figure 5a,b). With forskolin treatment, non‐CF organoids swelled, while CF organoids did not change in size (Figure 5c). During our studies, we noticed that forskolin treatment in some donors caused organoids to swell until a maximal point—they subsequently would “burst” and reduce in size. Consequently, to quantify the full effect of forskolin treatment, we measured the maximal swelling of organoids during the time course. We saw an exaggerated difference between non‐CF and CF organoid swelling (Figure 5d), suggesting that the effect of cAMP‐mediated secretion may be more than we initially observed. Collectively, these data suggest that CF intrahepatic cholangiocytes demonstrate morphological and fluid secretion defects.

## 4 DISCUSSION

We demonstrated that CF pig intrahepatic bile ducts have smaller duct lumens compared to non‐CF. We also show that CF pig intrahepatic cholangiocyte organoids are smaller at baseline and do not swell in response to cAMP‐mediated stimulation. These findings were observed in the absence of significant changes in serum‐based indicators of hepatobiliary disease. Therefore, these data suggest that defects in bile duct morphology and fluid secretion are present without significant clinically evident intrahepatic disease in newborn CF pigs.

These studies suggest that bile flow within the CF liver is decreased. Previous studies by our group demonstrate decreased secretin‐stimulated biliary flow into the intestine of newborn CF pigs (Uc et al., 2012). Our histological studies suggest that this reduced flow is correlated with smaller size of intrahepatic bile ducts. Studies suggest that people with CF have abnormalities with intrahepatic bile ducts; these studies demonstrated a wide variety of bile duct appearances including narrowing and tapering of some bile ducts but beading and dilation of others (Gaskin et al., 1988; King et al., 2000; Nagel et al., 1989). These latter changes may suggest luminal obstructions that dilate proximal portions of the biliary tract due to the accumulation of hepatic products and bile. Previous studies by our group and others also demonstrate narrowing in the extrahepatic biliary tract (Gaskin et al., 1988; Meyerholz et al., 2010; Nagel et al., 1989). In addition to changes in bile duct size, our data demonstrate a trend for greater mucus in CF intrahepatic bile ducts and this potentially could be a contributor to decreased bile flow in the newborn CF pig biliary tract. Further studies of the CF pig may aid in understanding this manifestation of CF hepatobiliary disease.

Our studies also found that CF intrahepatic biliary organoids had defective fluid secretion. This is consistent with our studies in the CF pig gallbladder (Zarei et al., 2020). The exact function of biliary fluid secretion, specifically pertaining to CFTR, is still not clear, but likely contributes to decreased bile flow in CF pigs (Uc et al., 2012). Our histology studies may suggest two possible roles. First, the trend of increased luminal mucus in CF pig intrahepatic bile ducts suggests that CFTR‐mediated fluid secretion may be important for mucus and bile clearance in the biliary tract. Previous studies have found bile plugs present in a minority of children with CF, which may represent the same mucus obstruction present in CF pig bile ducts (Lindblad et al., 1992; Lykavieris et al., 1996). This may be due to mucus accumulation due to decreased fluid secretion. Mice under chronic cholate feeding also demonstrated decreased biliary flow with CFTR loss (Bodewes et al., 2015a). In vitro studies with human induced pluripotent stem, cell‐derived cholangiocytes have also shown decreased secretin‐mediated fluid secretion with CFTR dysfunction (Fiorotto et al., 2018). Second, the structural changes of intrahepatic bile ducts in CF pigs imply that CFTR may be involved in the development of the biliary tract in utero. Studies in the pig airway demonstrate that CFTR loss contributes to airway developmental abnormalities (Meyerholz et al., 2018b).

Our previous studies in the CF pig gallbladder demonstrate fluid secretion defects. We found similar results here in CF pig intrahepatic bile ducts. These studies together may suggest a general mechanism of CF pathogenesis within the entire biliary tract: CFTR dysfunction leads to defective fluid secretion ultimately decreasing bile flow. The buildup of biliary luminal contents, over time, may contribute to CF hepatobiliary disease and ultimately end‐stage liver disease or cirrhosis.

Unlike our previous studies in the pig gallbladder, the difference in luminal mucus between non‐CF and CF in intrahepatic bile ducts was less dramatic. One possibility may be that CFTR is expressed at a greater level in the gallbladder relative to the intrahepatic bile ducts. It is possible that intrahepatic bile duct contents, including mucus, may be less reliant on CFTR for normal clearance. Another potential explanation for this may be that the gallbladder has a higher expression of gel‐forming mucins than the intrahepatic bile ducts at birth. Last, intrahepatic bile ducts may develop mucus accumulation and obstruction in advanced CF hepatobiliary disease. In any case, further studies of mucus accumulation in intrahepatic ducts are warranted.

Direct bilirubin, a biliary specific marker, was increased in CF pigs. One potential explanation for this may be mucus obstruction and decreased bile flow in the biliary tract—buildup within the bile ducts may ultimately spillover into the serum. The elevated direct hyperbilirubinemia in newborn CF pigs is the reminiscent of neonatal cholestasis, a rare and early form of CFLD (Leeuwen et al., 2014; Lindblad et al., 1999). Unlike CF pigs, however, CF neonatal cholestasis is typically self‐resolving without further complication and there are no clear associated histological changes (Leeuwen et al., 2014; Lindblad et al., 1999).

Our studies demonstrated that AST but not ALT levels were elevated in CF pigs. This may suggest an extrahepatic source of AST which can be found in other tissues including skeletal muscle, kidneys, and heart (Uhlen et al., 2015). Other studies have found cardiac and skeletal muscle dysfunction with CFTR defects (Gruet et al., 2017; Labombarda et al., 2016). These other results may suggest a mild cardiac and/or skeletal muscle phenotype in the CF pig that contributes to the elevated AST level.

Our current studies found that other serum‐based markers for hepatobiliary disease were not significantly elevated from non‐CF. People with CF demonstrate periodic increases in serum‐markers of hepatobiliary disease (Bodewes et al., 2015b; Woodruff et al., 2017). One study suggests that it is common for children with CF to have increased serum markers of hepatobiliary disease by age 21 (Woodruff et al., 2017). A possible explanation for the lack of significant changes in serum‐based markers in the current study may be that significant hepatobiliary disease may only be present in older CF animals. Due to challenges in defining and diagnosing CFLD, approximate age of onset is difficult to ascertain. Some studies have reported the average age of onset being between 5 and 8 years old (Colombo et al., 2002; Lindblad et al., 1999). Recent studies have indicated that CFLD is diagnosed more as people with CF get older (Boelle et al., 2019; Koh et al., 2017; Toledano et al., 2019). Regardless, in humans, neonatal presentation of CFLD is rare and hepatobiliary dysfunction primarily presents over several years (Colombo et al., 2002; Lindblad et al., 1999).

It is also possible that a second injury, in addition to CFTR loss, is required to initiate CFLD in pigs. Some mouse models of CFLD require the induction of chemical colitis in *CFTR* ^−/−^ mice (Fiorotto et al., 2011, 2016). Human cohort studies have been unsuccessful at identifying factors that explain why some individuals with CF develop liver disease while others do not (Bartlett et al., 2009; Boelle et al., 2019; Colombo et al., 2002; Lindblad et al., 1999). Additionally, attempts to identify modifier genes involved in CFLD pathogenesis have not been successful (Debray et al., 2019). One study found an association with the *SERPINA1 Z* allele and CFLD (Bartlett et al., 2009). Longitudinal studies with CF pigs may help better understand the time course and pathogenesis of CFLD. We have observed that some older CF pigs develop multilobular cirrhosis (Stoltz et al., 2013).

This study has strengths: (1) we used an animal model that resembles human CF disease; (2) we used clinical and histological measures to assess CFLD in a pig model; (3) we used a novel organoid model to study fluid secretion; (4) we assessed the markers of hepatobiliary disease at birth prior to the development of advanced disease. This study also has limitations: (1) the CF pig model may exaggerate certain features of CF hepatobiliary disease. For instance, microgallbladder occurs in up to 30% of people with CF; however, every newborn CF pig has some degree of microgallbladder. This complete penetrance of CF disease in the biliary tract may suggest that CFTR plays a more significant role in the porcine hepatobiliary tract relative to humans; (2) we did not conduct studies in older animals and did not address whether hepatobiliary disease is progressive in the CF pig; (3) we did not determine how CFTR loss contributes to findings in advanced disease, such as cirrhosis; (4) we do not have an appropriate and efficient methodology to comprehensively evaluate the entire intrahepatic biliary tract; (5) in addition to the intrahepatic changes presented in this study, CF pigs demonstrate extrahepatic disease and meconium ileus—it is challenging to distinguish how these other aspects of CF disease may impact the results of this study.

In this study, we determined that CF pigs have smaller intrahepatic bile ducts with fluid secretion defects at birth. These changes are present without significant elevation in serum markers of hepatobiliary disease relative to non‐CF. These findings suggest that decreased biliary clearance may be involved in the early pathogenesis of CF pig hepatobiliary disease.

## CONFLICT OF INTEREST

The University of Iowa Research Foundation has licensed materials and technologies related to CF pigs to Exemplar Genetics. DAS is a co‐inventor of CF pigs.

## AUTHOR CONTRIBUTIONS

KZ, DKM, DAS conceived and designed the research; KZ, DKM performed the experiments; KZ, DKM, DAS analyzed the data; KZ, DKM, DAS interpreted the results of experiments; KZ, DKM, DAS prepared the figures; KZ, DAS drafted the manuscript; KZ, DKM, DAS edited and revised the manuscript; KZ, DKM, DAS approved the final version of the manuscript.

## References

[phy214978-bib-0001] Bartlett, J. R. , Friedman, K. J. , Ling, S. C. , Pace, R. G. , Bell, S. C. , Bourke, B. , Castaldo, G. , Castellani, C. , Cipolli, M. , Colombo, C. , Colombo, J. L. , Debray, D. , Fernandez, A. , Lacaille, F. , Macek, M. Jr , Rowland, M. , Salvatore, F. , Taylor, C. J. , Wainwright, C. , Wilschanski, M. , Zemková, D. , Hannah, W. B. , Phillips, M. J. , Corey, M. , Zielenski, J. , Dorfman, R. , Wang, Y. , Zou, F. , Silverman, L. M. , Drumm, M. L. , Wright, F. A. , Lange, E. M. , Durie, P. R. , Knowles, M. R. , & Gene Modifier Study Group . (2009). Genetic modifiers of liver disease in cystic fibrosis. JAMA, 302(10), 1076–1083. 10.1001/jama.2009.1295. 19738092 PMC3711243

[phy214978-bib-0002] Blanc, W. A. , & Di Sant'Agnese, P. A. (1956). A distinctive type of biliary cirrhosis of the liver associated with cystic fibrosis of the pancreas; recognition through signs of portal hypertension. Pediatrics, 18(3), 387–409. 13359058

[phy214978-bib-0003] Bodewes, F. A. , Bijvelds, M. J. , de Vries, W. , Baller, J. F. , Gouw, A. S. , de Jonge, H. R. , & Verkade, H. J. (2015). Cholic acid induces a Cftr dependent biliary secretion and liver growth response in mice. PLoS One, 10(2), e0117599. 10.1371/journal.pone.0117599 25680200 PMC4334531

[phy214978-bib-0004] Bodewes, F. A. , van der Doef, H. P. , Houwen, R. H. , & Verkade, H. J. (2015). Increase of serum gamma‐glutamyltransferase associated with development of cirrhotic cystic fibrosis liver disease. Journal of Pediatric Gastroenterology and Nutrition, 61(1), 113–118. 25658056 10.1097/MPG.0000000000000758

[phy214978-bib-0005] Boelle, P. Y. , Debray, D. , Guillot, L. , Clement, A. , Corvol, H. , & French CF Modifier Gene Study Investigators . (2019). Cystic fibrosis liver disease: outcomes and risk factors in a large cohort of French patients. Hepatology, 69(4), 1648–1656. 10.1002/hep.30148. 30058245 PMC6519059

[phy214978-bib-0006] Boj, S. F. , Vonk, A. M. , Statia, M. , Su, J. , Vries, R. R. , Beekman, J. M. , & Clevers, H. (2017). Forskolin‐induced swelling in intestinal organoids: An in vitro assay for assessing drug response in cystic fibrosis patients. Journal of Visualized Experiments, 120, 55159. 10.3791/55159 PMC5408767 28287550

[phy214978-bib-0007] Cohn, J. A. , Strong, T. V. , Picciotto, M. R. , Nairn, A. C. , Collins, F. S. , & Fitz, J. G. (1993). Localization of the cystic fibrosis transmembrane conductance regulator in human bile duct epithelial cells. Gastroenterology, 105(6), 1857–1864. 10.1016/0016-5085(93)91085-V. 7504645

[phy214978-bib-0008] Colombo, C. (2007). Liver disease in cystic fibrosis. Current Opinion in Pulmonary Medicine, 13(6), 529–536. 10.1097/MCP.0b013e3282f10a16. 17901760

[phy214978-bib-0009] Colombo, C. , Battezzati, P. M. , Crosignani, A. , Morabito, A. , Costantini, D. , Padoan, R. , & Giunta, A. (2002). Liver disease in cystic fibrosis: a prospective study on incidence, risk factors, and outcome. Hepatology, 36(6), 1374–1382. 10.1002/hep.1840360613. 12447862

[phy214978-bib-0010] Debray, D. , Corvol, H. , & Housset, C. (2019). Modifier genes in cystic fibrosis‐related liver disease. Current Opinion in Gastroenterology, 35(2), 88–92. 10.1097/MOG.0000000000000508. 30585791

[phy214978-bib-0011] Debray, D. , Kelly, D. , Houwen, R. , Strandvik, B. , & Colombo, C. (2011). Best practice guidance for the diagnosis and management of cystic fibrosis‐associated liver disease. Journal of Cystic Fibrosis, 10(suppl 2), S29–S36. 10.1016/S1569-1993(11)60006-4. 21658639

[phy214978-bib-0012] Debray, D. , Rainteau, D. , Barbu, V. , Rouahi, M. , El Mourabit, H. , Lerondel, S. , Rey, C. , Humbert, L. , Wendum, D. , Cottart, C.–H. , Dawson, P. , Chignard, N. , & Housset, C. (2012). Defects in gallbladder emptying and bile acid homeostasis in mice with cystic fibrosis transmembrane conductance regulator deficiencies. Gastroenterology, 142(7), 1581–91 e6. 10.1053/j.gastro.2012.02.033. 22370478 PMC3579557

[phy214978-bib-0013] Dekkers, J. F. , Alieva, M. , Wellens, L. M. , Ariese, H. C. R. , Jamieson, P. R. , Vonk, A. M. , Amatngalim, G. D. , Hu, H. , Oost, K. C. , Snippert, H. J. G. , Beekman, J. M. , Wehrens, E. J. , Visvader, J. E. , Clevers, H. , & Rios, A. C. (2019). High‐resolution 3D imaging of fixed and cleared organoids. Nature Protocols, 14(6), 1756–1771. 10.1038/s41596-019-0160-8. 31053799

[phy214978-bib-0014] Fiorotto, R. , Amenduni, M. , Mariotti, V. , Cadamuro, M. , Fabris, L. , Spirli, C. , & Strazzabosco, M. (2019). Animal models for cystic fibrosis liver disease (CFLD). Biochimica et Biophysica Acta Molecular Basis of Disease, 1865(5), 965–969. 10.1016/j.bbadis.2018.07.026. 30071276 PMC6474816

[phy214978-bib-0015] Fiorotto, R. , Amenduni, M. , Mariotti, V. , Fabris, L. , Spirli, C. , & Strazzabosco, M. (2018). Src kinase inhibition reduces inflammatory and cytoskeletal changes in DeltaF508 human cholangiocytes and improves cystic fibrosis transmembrane conductance regulator correctors efficacy. Hepatology, 67(3), 972–988. 28836688 10.1002/hep.29400 PMC5783790

[phy214978-bib-0016] Fiorotto, R. , Scirpo, R. , Trauner, M. , Fabris, L. , Hoque, R. , Spirli, C. , & Strazzabosco, M. (2011). Loss of CFTR affects biliary epithelium innate immunity and causes TLR4‐NF‐kappaB‐mediated inflammatory response in mice. Gastroenterology, 141(4), 1498–1508, 508 e1–5. 21712022 10.1053/j.gastro.2011.06.052 PMC3186841

[phy214978-bib-0017] Fiorotto, R. , Villani, A. , Kourtidis, A. , Scirpo, R. , Amenduni, M. , Geibel, P. J. , Cadamuro, M. , Spirli, C. , Anastasiadis, P. Z. , & Strazzabosco, M. (2016). The cystic fibrosis transmembrane conductance regulator controls biliary epithelial inflammation and permeability by regulating Src tyrosine kinase activity. Hepatology, 64(6), 2118–2134. 10.1002/hep.28817. 27629435 PMC5115965

[phy214978-bib-0018] Foundation CF . (2019). Cystic fibrosis foundation patient registry highlights. Cystic Fibrosis Foundation. https://www.cff.org/Research/Researcher‐Resources/Patient‐Registry/Cystic‐Fibrosis‐Foundation‐Patient‐Registry‐Highlights.pdf.

[phy214978-bib-0019] Gaskin, K. J. , Waters, D. L. , Howman‐Giles, R. , de Silva, M. , Earl, J. W. , Martin, H. C. , Kan, A. E. , Brown, J. M. , & Dorney, S. F. A. (1988). Liver disease and common‐bile‐duct stenosis in cystic fibrosis. New England Journal of Medicine, 318(6), 340–346. 10.1056/NEJM198802113180602. 3340104

[phy214978-bib-0020] Gruet, M. , Troosters, T. , & Verges, S. (2017). Peripheral muscle abnormalities in cystic fibrosis: etiology, clinical implications and response to therapeutic interventions. Journal of Cystic Fibrosis, 16(5), 538–552. 10.1016/j.jcf.2017.02.007. 28262570

[phy214978-bib-0021] King, L. J. , Scurr, E. D. , Murugan, N. , Williams, S. G. , Westaby, D. , & Healy, J. C. (2000). Hepatobiliary and pancreatic manifestations of cystic fibrosis: MR imaging appearances. Radiographics, 20(3), 767–777. 10.1148/radiographics.20.3.g00ma08767. 10835127

[phy214978-bib-0022] Koh, C. , Sakiani, S. , Surana, P. , Zhao, X. , Eccleston, J. , Kleiner, D. E. , Herion, D. , Liang, T. J. , Hoofnagle, J. H. , Chernick, M. , & Heller, T. (2017). Adult‐onset cystic fibrosis liver disease: Diagnosis and characterization of an underappreciated entity. Hepatology, 66(2), 591–601. 10.1002/hep.29217. 28422310 PMC5519421

[phy214978-bib-0023] Labombarda, F. , Saloux, E. , Brouard, J. , Bergot, E. , & Milliez, P. (2016). Heart involvement in cystic fibrosis: A specific cystic fibrosis‐related myocardial changes? Respiratory Medicine, 118, 31–38. 10.1016/j.rmed.2016.07.011. 27578468

[phy214978-bib-0024] Lamireau, T. , Monnereau, S. , Martin, S. , Marcotte, J. E. , Winnock, M. , & Alvarez, F. (2004). Epidemiology of liver disease in cystic fibrosis: A longitudinal study. Journal of Hepatology, 41(6), 920–925. 10.1016/j.jhep.2004.08.006. 15582124

[phy214978-bib-0025] Leeuwen, L. , Magoffin, A. K. , Fitzgerald, D. A. , Cipolli, M. , & Gaskin, K. J. (2014). Cholestasis and meconium ileus in infants with cystic fibrosis and their clinical outcomes. Archives of Disease in Childhood, 99(5), 443–447. 10.1136/archdischild-2013-304159. 24436365

[phy214978-bib-0026] Leung, D. H. , & Narkewicz, M. R. (2017). Cystic fibrosis‐related cirrhosis. Journal of Cystic Fibrosis, 16(suppl 2), S50–S61. 10.1016/j.jcf.2017.07.002. 28986027

[phy214978-bib-0027] Lindblad, A. , Glaumann, H. , & Strandvik, B. (1999). Natural history of liver disease in cystic fibrosis. Hepatology, 30(5), 1151–1158. 10.1002/hep.510300527. 10534335

[phy214978-bib-0028] Lindblad, A. , Hultcrantz, R. , & Strandvik, B. (1992). Bile‐duct destruction and collagen deposition: A prominent ultrastructural feature of the liver in cystic fibrosis. Hepatology, 16(2), 372–381. 10.1002/hep.1840160215. 1639346

[phy214978-bib-0029] Lykavieris, P. , Bernard, O. , & Hadchouel, M. (1996). Neonatal cholestasis as the presenting feature in cystic fibrosis. Archives of Disease in Childhood, 75(1), 67–70. 10.1136/adc.75.1.67. 8813874 PMC1511658

[phy214978-bib-0030] Meyerholz, D. K. , & Beck, A. P. (2018). Principles and approaches for reproducible scoring of tissue stains in research. Laboratory Investigation, 98(7), 844–855. 10.1038/s41374-018-0057-0. 29849125

[phy214978-bib-0031] Meyerholz, D. K. , Beck, A. P. , Goeken, J. A. , Leidinger, M. R. , Ofori‐Amanfo, G. K. , Brown, H. C. , Businga, T. R. , Stoltz, D. A. , Reznikov, L. R. , & Flaherty, H. A. (2018). Glycogen depletion can increase the specificity of mucin detection in airway tissues. BMC Research Notes, 11(1), 763. 10.1186/s13104-018-3855-y. 30359291 PMC6203197

[phy214978-bib-0032] Meyerholz, D. K. , Lambertz, A. M. , Reznikov, L. R. , Ofori‐Amanfo, G. K. , Karp, P. H. , McCray, P. B. Jr , Welsh, M. J. , & Stoltz, D. A. (2016). Immunohistochemical detection of markers for translational studies of lung disease in pigs and humans. Toxicologic Pathology, 44(3), 434–441. 10.1177/0192623315609691. 26511846 PMC4805467

[phy214978-bib-0033] Meyerholz, D. K. , Stoltz, D. A. , Gansemer, N. D. , Ernst, S. E. , Cook, D. P. , Strub, M. D. , LeClair, E. N. , Barker, C. K. , Adam, R. J. , Leidinger, M. R. , Gibson‐Corley, K. N. , Karp, P. H. , Welsh, M. J. , & McCray, P. B. (2018). Lack of cystic fibrosis transmembrane conductance regulator disrupts fetal airway development in pigs. Laboratory Investigation, 98(6), 825–838. 10.1038/s41374-018-0026-7. 29467455 PMC6019641

[phy214978-bib-0034] Meyerholz, D. K. , Stoltz, D. A. , Pezzulo, A. A. , & Welsh, M. J. (2010). Pathology of gastrointestinal organs in a porcine model of cystic fibrosis. American Journal of Pathology, 176(3), 1377–1389. 10.2353/ajpath.2010.090849. PMC2832157 20110417

[phy214978-bib-0035] Nagel, R. A. , Westaby, D. , Javaid, A. , Kavani, J. , Meire, H. B. , Lombard, M. G. , Williams, R. , & Hodson, M. (1989). Liver disease and bile duct abnormalities in adults with cystic fibrosis. Lancet, 2(8677), 1422–1425. 10.1016/S0140-6736(89)92035-7. 2574362

[phy214978-bib-0036] Nieto‐Torres, J. L. , DeDiego, M. L. , Verdia‐Baguena, C. , Jimenez‐Guardeno, J. M. , Regla‐Nava, J. A. , Fernandez‐Delgado, R. , Castaño‐Rodriguez, C. , Alcaraz, A. , Torres, J. , Aguilella, V. M. , & Enjuanes, L. (2014). Severe acute respiratory syndrome coronavirus envelope protein ion channel activity promotes virus fitness and pathogenesis. PLoS Pathogens, 10(5), e1004077. 10.1371/journal.ppat.1004077. 24788150 PMC4006877

[phy214978-bib-0037] Olivier, A. K. , Gibson‐Corley, K. N. , & Meyerholz, D. K. (2015). Animal models of gastrointestinal and liver diseases. Animal models of cystic fibrosis: Gastrointestinal, pancreatic, and hepatobiliary disease and pathophysiology. American Journal of Physiology‐Gastrointestinal and Liver Physiology, 308(6), G459–G471. 10.1152/ajpgi.00146.2014. 25591863 PMC4360044

[phy214978-bib-0038] Oppenheimer, E. H. , & Esterly, J. R. (1975). Hepatic changes in young infants with cystic fibrosis: Possible relation to focal biliary cirrhosis. Journal of Pediatrics, 86(5), 683–689. 10.1016/S0022-3476(75)80351-9. 1133649

[phy214978-bib-0039] Oppenheimer, E. H. , & Esterly, J. R. (1975). Pathology of cystic fibrosis review of the literature and comparison with 146 autopsied cases. Perspectives in Pediatric Pathology, 2, 241–278. 1168897

[phy214978-bib-0040] Registry CFFP . (2018). Annual data report. Cystic Fibrosis Foundation; 2019. https://www.cff.org/Research/Researcher‐Resources/Patient‐Registry/2018‐Patient‐Registry‐Annual‐Data‐Report.pdf.

[phy214978-bib-0041] Rogers, C. S. , Stoltz, D. A. , Meyerholz, D. K. , Ostedgaard, L. S. , Rokhlina, T. , Taft, P. J. , Rogan, M. P. , Pezzulo, A. A. , Karp, P. H. , Itani, O. A. , Kabel, A. C. , Wohlford‐Lenane, C. l. , Davis, G. J. , Hanfland, R. A. , Smith, T. l. , Samuel, M. , Wax, D. , Murphy, C. N. , Rieke, A. , Whitworth, K. , Uc, A. , Starner, T. D. , Brogden, K. A. , Shilyansky, J. , McCray, P. B. , Zabner, J. , Prather, R. S. , & Welsh, M. J. (2008). Disruption of the CFTR gene produces a model of cystic fibrosis in newborn pigs. Science, 321(5897), 1837–1841. 10.1126/science.1163600. 18818360 PMC2570747

[phy214978-bib-0042] Sampaziotis, F. , Justin, A. W. , Tysoe, O. C. , Sawiak, S. , Godfrey, E. M. , Upponi, S. S. , Gieseck, R. L. , de Brito, M. C. , Berntsen, N. L. , Gómez‐Vázquez, M. J. , Ortmann, D. , Yiangou, L. , Ross, A. , Bargehr, J. , Bertero, A. , Zonneveld, M. C. F. , Pedersen, M. T. , Pawlowski, M. , Valestrand, L. , Madrigal, P. , Georgakopoulos, N. , Pirmadjid, N. , Skeldon, G. M. , Casey, J. , Shu, W. , Materek, P. M. , Snijders, K. E. , Brown, S. E. , Rimland, C. A. , Simonic, I. , Davies, S. E. , Jensen, K. B. , Zilbauer, M. , Gelson, W. T. H. , Alexander, G. J. , Sinha, S. , Hannan, N. R. F. , Wynn, T. A. , Karlsen, T. H. , Melum, E. , Markaki, A. E. , Saeb‐Parsy, K. , & Vallier, L. (2017). Reconstruction of the mouse extrahepatic biliary tree using primary human extrahepatic cholangiocyte organoids. Nature Medicine, 23(8), 954–963. 10.1038/nm.4360. 28671689

[phy214978-bib-0043] Stoltz, D. A. , Rokhlina, T. , Ernst, S. E. , Pezzulo, A. A. , Ostedgaard, L. S. , Karp, P. H. , Samuel, M. S. , Reznikov, L. R. , Rector, M. V. , Gansemer, N. D. , Bouzek, D. C. , Alaiwa, M. H. A. , Hoegger, M. J. , Ludwig, P. S. , Taft, P. J. , Wallen, T. J. , Wohlford‐Lenane, C. , McMenimen, J. D. , Chen, J.‐H. , Bogan, K. L. , Adam, R. J. , Hornick, E. E. , Nelson, G. A. , Hoffman, E. A. , Chang, E. H. , Zabner, J. , McCray, P. B. , Prather, R. S. , Meyerholz, D. K. , & Welsh, M. J. (2013). Intestinal CFTR expression alleviates meconium ileus in cystic fibrosis pigs. Journal of Clinical Investigation, 123(6), 2685–2693. 10.1172/JCI68867. PMC3668832 23676501

[phy214978-bib-0044] Toledano, M. B. , Mukherjee, S. K. , Howell, J. , Westaby, D. , Khan, S. A. , Bilton, D. , & Simmonds, N. J. (2019). The emerging burden of liver disease in cystic fibrosis patients: A UK nationwide study. PLoS One, 14(4), e0212779. 10.1371/journal.pone.0212779. 30947265 PMC6448894

[phy214978-bib-0045] Tysoe, O. C. , Justin, A. W. , Brevini, T. , Chen, S. E. , Mahbubani, K. T. , Frank, A. K. , Zedira, H. , Melum, E. , Saeb‐Parsy, K. , Markaki, A. E. , Vallier, L. , & Sampaziotis, F. (2019). Isolation and propagation of primary human cholangiocyte organoids for the generation of bioengineered biliary tissue. Nature Protocols, 14(6), 1884–1925. 10.1038/s41596-019-0168-0. 31110298

[phy214978-bib-0046] Uc, A. , Giriyappa, R. , Meyerholz, D. K. , Griffin, M. , Ostedgaard, L. S. , Tang, X. X. , Abu‐El‐Haija, M. , Stoltz, D. A. , Ludwig, P. , Pezzulo, A. , Abu‐El‐Haija, M. , Taft, P. , & Welsh, M. J. (2012). Pancreatic and biliary secretion are both altered in cystic fibrosis pigs. American Journal of Physiology‐Gastrointestinal and Liver Physiology, 303(8), G961–G968. 10.1152/ajpgi.00030.2012. 22936270 PMC3469695

[phy214978-bib-0047] Uhlen, M. , Fagerberg, L. , Hallstrom, B. M. , Lindskog, C. , Oksvold, P. , Mardinoglu, A. , Sivertsson, A. , Kampf, C. , Sjostedt, E. , Asplund, A. , Olsson, I. , Edlund, K. , Lundberg, E. , Navani, S. , Szigyarto, C. A.‐K , Odeberg, J. , Djureinovic, D. , Takanen, J. O. , Hober, S. , Alm, T. , Edqvist, P‐H , Berling, H. , Tegel, H. , Mulder, J. , Rockberg, J. , Nilsson, P. , Schwenk, J. M , Hamsten, M. , von Feilitzen, K. , Forsberg, M. , Persson, L. , Johansson, F. , Zwahlen, M. , von Heijne, G. , Nielsen, J. , & Ponten, F. (2015). Proteomics. Tissue‐based map of the human proteome. Science, 347, 1260419. 10.1126/science.1260419. 25613900

[phy214978-bib-0048] Vawter, G. F. , & Shwachman, H. (1979). Cystic fibrosis in adults: An autopsy study. Pathology Annual, 14(Pt 2), 357–382. 547223

[phy214978-bib-0049] Woodruff, S. A. , Sontag, M. K. , Accurso, F. J. , Sokol, R. J. , & Narkewicz, M. R. (2017). Prevalence of elevated liver enzymes in children with cystic fibrosis diagnosed by newborn screen. Journal of Cystic Fibrosis, 16(1), 139–145. 10.1016/j.jcf.2016.08.002. 27555301

[phy214978-bib-0050] Zarei, K. , Stroik, M. R. , Gansemer, N. D. , Thurman, A. L. , Ostedgaard, L. S. , Ernst, S. E. , Thornell, I. M. , Powers, L. S. , Pezzulo, A. A. , Meyerholz, D. K. , & Stoltz, D. A. (2020). Early pathogenesis of cystic fibrosis gallbladder disease in a porcine model. Laboratory Investigation, 100(11), 1388–1399. 10.1038/s41374-020-0474-8. 32719544 PMC7578062

